# Peptide Lv Promotes Trafficking and Membrane Insertion of K_Ca_3.1 through the MEK1–ERK and PI3K–Akt Signaling Pathways

**DOI:** 10.3390/cells12121651

**Published:** 2023-06-17

**Authors:** Dylan L. Pham, Autumn Niemi, Ria Blank, Gabriella Lomenzo, Jenivi Tham, Michael L. Ko, Gladys Y.-P. Ko

**Affiliations:** 1 Department of Veterinary Integrative Biosciences, School of Veterinary Medicine and Biomedical Sciences, Texas A&M University, College Station, TX 77843, USA; phamdarch@tamu.edu (D.L.P.); autumnniemi@tamu.edu (A.N.); kmblank246@tamu.edu (R.B.); g_lomenzo11@tamu.edu (G.L.); jenivit@tamu.edu (J.T.); michael.ko@blinn.edu (M.L.K.); 2 Department of Biology, Division of Natural and Physical Sciences, Blinn College, Bryan, TX 77802, USA; 3 Texas A&M Institute for Neuroscience, Texas A&M University, College Station, TX 77843, USA

**Keywords:** angiogenesis, potassium channel, endothelial cell, peptide Lv, protein trafficking, signaling pathway

## Abstract

Peptide Lv is a small endogenous secretory peptide that is proangiogenic through hyperpolarizing vascular endothelial cells (ECs) by enhancing the current densities of K_Ca_3.1 channels. However, it is unclear how peptide Lv enhances these currents. One way to enhance the current densities of ion channels is to promote its trafficking and insertion into the plasma membrane. We hypothesized that peptide Lv-elicited K_Ca_3.1 augmentation occurs through activating the mitogen-activated protein kinase kinase 1 (MEK1)-extracellular signal-regulated kinase (ERK) and phosphoinositide 3-kinase (PI3K)–protein kinase B (Akt) signaling pathways, which are known to mediate ion channel trafficking and membrane insertion in neurons. To test this hypothesis, we employed patch-clamp electrophysiological recordings and cell-surface biotinylation assays on ECs treated with peptide Lv and pharmaceutical inhibitors of ERK and Akt. Blocking ERK or Akt activation diminished peptide Lv-elicited EC hyperpolarization and increase in K_Ca_3.1 current densities. Blocking PI3K or Akt activation decreased the level of plasma membrane-bound, but not the total amount of K_Ca_3.1 protein in ECs. Therefore, the peptide Lv-elicited EC hyperpolarization and K_Ca_3.1 augmentation occurred in part through channel trafficking and insertion mediated by MEK1–ERK and PI3K–Akt activation. These results demonstrate the molecular mechanisms of how peptide Lv promotes EC-mediated angiogenesis.

## 1. Introduction

Peptide Lv is a newly discovered endogenous secretory peptide with about 40–50 amino acids depending on species, is highly conserved across species [1], and is expressed in various organs and tissues including the retina and vascular endothelium [1,2]. Functionally, peptide Lv is able to augment ion channel currents in neurons, cardiomyocytes, and vascular endothelial cells (ECs) [1,2,3,4], as it was first discovered to augment L-type voltage-gated calcium channels in retinal photoreceptors [1,2], thus the name peptide “Lv”. Peptide Lv is also proangiogenic through stimulating EC proliferation, migration, and sprouting, and it can elicit vasodilation [2,3]. Peptide Lv is upregulated in the retinas of patients with early proliferative diabetic retinopathy, diabetic animals, and mice with oxygen-induced retinopathy [3]. Thus, peptide Lv may contribute to pathological neovascularization.

The angiogenic activity of peptide Lv occurs in part through its ability to bind and activate vascular endothelial growth factor receptor 2 (VEGFR2) [2,3]. It also resembles VEGF and elicits vasodilation of coronary and retinal arterioles [3,5]. VEGF-elicited vasodilation is dependent on nitric oxide (NO), since N(G)-nitro-L-arginine methyl ester (L-NAME), a NO synthase inhibitor, completely blocks VEGF-elicited vasodilation [5]. However, peptide Lv-elicited vasodilation is only partially blocked by L-NAME [3], indicating that peptide Lv has a VEGF/VEGFR2-independent mechanism for vasodilation and possibly for angiogenesis. The hyperpolarization of ECs in blood vessels can lead to vasodilation and angiogenesis, which can be independent of NO [6,7,8,9,10]. Opening of the potassium channels mediates EC hyperpolarization, leading to smooth muscle cell hyperpolarization and relaxation, which dilates blood vessels [6,7,8,11,12]. Calcium-dependent potassium (K_Ca_) channels, in particular the small-conductance K_Ca_ (K_Ca_2.3) and intermediate-conductance K_Ca_ (K_Ca_3.1) channels, are two of the major potassium channels involved in EC hyperpolarization [8,13,14].

We recently showed that peptide Lv hyperpolarizes cultured ECs by augmenting K_Ca_3.1, and blocking K_Ca_3.1 prevents peptide Lv-elicited proliferation of ECs [4]. This finding suggests that K_Ca_3.1 may play a role in VEGF-independent angiogenesis, as EC proliferation is a major process in angiogenesis [15,16,17]. We found that peptide Lv increases the expression of K_Ca_3.1 [4], but increased ion channel expression in itself does not necessarily increase the channel’s current densities [18,19,20]. Ion channel proteins must be properly shuttled and inserted into the plasma membrane to be physiologically functional [18,19,20]. As peptide Lv augments the current density of K_Ca_3.1 in ECs, it is possible that peptide Lv promotes the trafficking and membrane insertion of K_Ca_3.1. In neurons, exogenous trophic factors increase K_Ca_ currents by increasing protein expression and promoting trafficking and membrane insertion of the channel complex [18,20]. The mitogen-activated protein kinase kinase (MEK1)-extracellular signal-regulated kinase (ERK) [20], and the phosphoinositide 3-kinase (PI3K)–protein kinase B (Akt) signaling pathways [18] are known to mediate ion channel trafficking. Peptide Lv stimulates the activation/phosphorylation of ERK in photoreceptors and cardiomyocytes [1,2]. Thus, we postulated that peptide Lv promotes trafficking and membrane insertion of K_Ca_3.1 in ECs in a similar manner. In this study, we employed patch-clamp recordings, biotinylation assays of plasma membrane-bound proteins, Western blots, and pharmacological tools to decipher the molecular signaling underlying the possibility that peptide Lv indeed promotes trafficking and plasma membrane insertion of K_Ca_3.1 in cultured ECs.

## 2. Materials and Methods

### 2.1. Chemicals

Peptide Lv was custom-made from Peptide 2.0 Inc. (Chantilly, VA, USA). The polyclone antibody specifically against peptide Lv, anti-Lv, was obtained from Biomatik (Cambridge, ON, Canada). Peptide Lv and anti-Lv were made using the murine amino acid sequence DSLLAVRWFFAPDGSQEALMVKMTKLRIIQYYGNFSRTANQQRLRLLEE [1,2,3]. Peptide Lv and anti-Lv tested negative for endotoxin.

Other inhibitors and chemicals used in this study were TRAM-34 (K_Ca_3.1 inhibitor; #AAJ60019-MB, Thermo Fisher Scientific, Waltham, MA, USA), β-escin (#E1378, Sigma-Aldrich, St. Louis, MO, USA), FR180402 (#SML0320, Sigma-Aldrich), PD98059 (#513000, Sigma-Aldrich), Akti (#A6730, Sigma-Aldrich), and LY294002 (#440202, Sigma-Aldrich).

### 2.2. Mice

C57BL/6J mice were originally purchased from the Jackson Laboratory (Bar Harbor, ME, USA), then bred and maintained at Texas A&M University. Mice were housed under temperature- and humidity-controlled conditions with 12:12 h light–dark cycles. Food and water were given ad libitum. All animal experiments were approved (AUP# 2020-0286) by the Institutional Animal Care and Use Committee of Texas A&M University.

### 2.3. Retinal Vasculature Immunofluorescent Staining

The fixation and trypsinization of the mouse retinas were processed as we previously described [21]. In brief, mouse eyes were collected and fixed with 4% paraformaldehyde at 4 °C overnight (for 20 h). Whole retinas were dissected and kept in double-deionized water with gentle rocking overnight at room temperature. The retinas were then incubated with 3% trypsin (#215250, BD Biosciences, Franklin Lakes, NJ, USA) for 1.5 h at 37 °C, for trypsinization followed by washing with double-deionized water by gentle pipetting to remove neural tissue. The remaining network of retinal vasculature was transferred to a glass slide and flattened (whole-mounted), blocked with 10% goat serum in phosphate-buffered saline (PBS) for 2 h at room temperature, and then incubated with primary antibodies overnight at 4 °C. After washing with PBS, the whole-mounted retinal vasculatures were incubated with secondary antibodies for 2 h at room temperature and mounted with ProLong Gold antifade with DAPI (#P36931, Invitrogen, Waltham, MA, USA). Images were obtained using a Zeiss Axiovert 200M microscope (Carl Zeiss AG, Jena, Germany) [21,22,23].

The following primary antibodies were used: anti-Lv (1:1000 dilution) and anti-CD31 (#3528, Cell Signaling Technology, Danvers, MA, USA). The following secondary antibodies were used: Fluor 488 goat anti-rabbit immunoglobulin G (IgG; 1:150 dilution; Thermo Fisher Scientific) and Cy5 goat anti-mouse IgG (1:150 dilution; Abcam, Cambridge, MA, USA).

### 2.4. Cell Culture

Cell cultures were maintained as we previously described [4]. Human umbilical vein ECs (HUVECs; #200-05n, Cell Applications Inc., San Diego, CA, USA) and human retinal microvascular ECs (HRMECs; #ACBRI 181, Cell Systems, Kirkland, WA, USA) were cultured in EGM-2 MV Microvascular Endothelial Cell Growth Medium-2 BulletKit (EGM; #CC-3202, Lonza, Walkersville, MD, USA) at 37 °C and 5% CO_2_.

### 2.5. Patch-Clamp Electrophysiology

The β-escin-based perforated patch method [24] was used with the whole-cell patch-clamp configuration on cultured HUVECs as we previously described [1,2]. The methods and parameters for recording endothelial membrane potentials (current-clamp) and the K_Ca_3.1 current (voltage-clamp) were based on our previous publications [1,4]. The external solution contained (in mM): 160 NaCl, 4.5 KCl, 1 MgCl_2_, 2 CaCl_2_, 10 HEPES, and 1 glucose, pH 7.5 adjusted with NaOH. The pipette solution contained (in mM): 120 KCl, 1.75 MgCl_2_, 1 Na_2_ATP, 10 EGTA, 4.1 CaCl_2_, and 10 HEPES, pH 7.2 adjusted with KOH. The free calcium concentration in the pipette solution was calculated to be 100 nM using an online calcium chelator calculator [15]. β-escin was freshly prepared as a 35 mM stock solution in double-deionized water, kept on ice, and then added to the pipette solution to yield a final concentration of 35 μM.

HUVECs were seeded onto 12 mm acid-washed glass coverslips and placed in the incubator for 48 h to allow for adhesion. HUVECs were treated with PBS (vehicle; control) or peptide Lv (500 ng/mL) and maintained in the incubator at 37 °C and 5% CO_2_ for 3 h prior to the patch-clamp recordings. There was no statistical difference in recorded amplitudes (either membrane potentials or currents) between the controls (either treated with PBS or without treatment), so their data were combined as a single control group. 

Recordings were performed as we described previously [4]. In brief, all recordings were performed at room temperature (23 °C) using an A-M 2400 amplifier (A-M Systems Inc., Carlsborg, WA, USA). Signals were low-pass filtered at 1 kHz and digitized at 5 kHz with a Digidata 1550A interface (Axon Instruments/Molecular Devices, Union City, CA, USA), and pCLAMP 10.0 software (Molecular Devices) was used for data acquisition and analysis. Electrode capacitance was compensated after gigaohm (GΩ) seals were formed. The membrane capacitance, series resistance, and input resistance of the recorded HUVECs were measured by applying a +5 mV (100 ms) depolarizing voltage step from a holding potential of −60 mV. Any cell with an input resistance <1 GΩ was discarded. The membrane capacitance reading was used as the value for the whole-cell capacitance. The outward currents were elicited with a step command from a holding potential at −60 mV to 40 mV for 300 ms, as this current step command elicited the peak outward current [4]. From the same cell, the total outward current containing K_Ca_3.1 was first recorded, then TRAM-34 (10 µM; K_Ca_3.1 inhibitor) was perfused into the recording chamber for 5 min, and then a second current elicited and recorded in the presence of TRAM-34. The K_Ca_3.1 current from a single cell was isolated by subtracting the current under TRAM-34 perfusion from the total outward current. The current density (pA/pF) was obtained by dividing the K_Ca_3.1 current amplitude (measured at 200 ms, the tau point) by the whole-cell capacitance. The membrane potentials were recorded under the current-clamp mode by injecting a 20 pA current for 750 ms. 

### 2.6. Immunoblotting

Western blots were performed as we previously described [4]. HUVECs and HRMECs were seeded onto 60 mm culture plates and grown to 100% confluency. Cultured HUVECs and HMRECs were then treated with PBS (vehicle control) or peptide Lv (500 ng/mL) for various times up to 3 h. Lysates were then collected for immunoblot analysis as we described previously [1,2,4]. In brief, cells were collected and lysed with an ice-cold RIPA lysis buffer, and proteins were denatured by mixing the lysate with 2X Lamelli sample buffer and heating for 5 min at 95 °C. Samples were separated using a 10% SDS-polyacrylamide gel and then transferred to a nitrocellulose membrane. Membranes were incubated with the primary antibodies K_Ca_3.1 (1:400; #LS-C171766-100; LSBio, Seattle, WA, USA), phospho-p44/42 MAPK (1:1000; Erk1/2, Thr202/Tyr204; #4370, Cell Signaling Technology), p44/42 (1:1000; pan-Erk1/2; #4695, Cell Signaling Technology), phospho-Akt (1:800; ser473; #4060, Cell Signaling Technology), pan-Akt (1:1000; #4691, Cell Signaling Technology), and β-actin (1:1000; #4970S, Cell Signaling Technologies) overnight at 4 °C. The membranes were then washed with a tris-base saline (TBS)–tween solution and incubated with anti-rabbit IgG, HRP-linked secondary antibody (1:1000; #7074S, Cell Signaling Technologies) for 1 h at room temperature (23 °C). The membranes were visualized using Super Signal West Pico or Femto chemiluminescent substrate kit (#34078 or #34096, Pierce Biotechnology Inc., Rockford, IL, USA) with an immunoblot scanner (LI-COR Biosciences, Lincoln, NE, USA). Band intensities were quantified using ImageJ software (https://imagej.nih.gov/ij/; National Institutes of Health; NIH, Bethesda, MA, USA). For K_Ca_3.1 analysis, the band intensities were first normalized to the internal control, β-actin, and the relative changes were quantified according to the method described by Janes [25]. The pERK and pAkt were normalized to total ERK and total Akt, respectively.

### 2.7. Cell-Surface Biotinylation Assay

Cultured HUVECs were treated with PBS (vehicle control) or peptide Lv (500 ng/mL) in the presence/absence of various inhibitors for 3 h. Biotinylation assays were performed as we described previously [26] and according to the company’s protocol. In short, cultures were incubated with EZ-Link™ Sulfo-NHS-LC-Biotin (#21335; Pierce Biotechnology) for 30 min at room temperature while being rocked and for 30 min at 4 °C while being rocked. The reaction was quenched with 100 mM glycine. Cells were lysed with a RIPA lysis buffer. A portion of the lysate was collected to test for total protein concentration. The remaining sample was incubated with streptavidin agarose beads (#20353; Pierce Biotechnology) for 1 h at 4 °C under rotation. The supernatant was collected and tested for cytoplasmic protein concentration using immunoblot assays as described above. Lamelli buffer was added to the beads, and the resulting mixture was heated at 95 °C for 5 min to dissociate the protein from the beads. 

### 2.8. Statistical Analysis

All data are presented as mean ± standard error of the mean (SEM). The comparisons between two groups were analyzed using Student’s *t* test. Differences between multiple groups were analyzed by one-way ANOVA and Tukey *post hoc* tests. Origin 8.6 (OriginLab, Northampton, MA, USA) was used for statistical analyses. Throughout, *p* < 0.05 was considered significant.

## 3. Results

### 3.1. Peptide Lv Augments K_Ca_3.1 Current Density and Endothelial Hyperpolarization through ERK Activation

**Figure 1:**
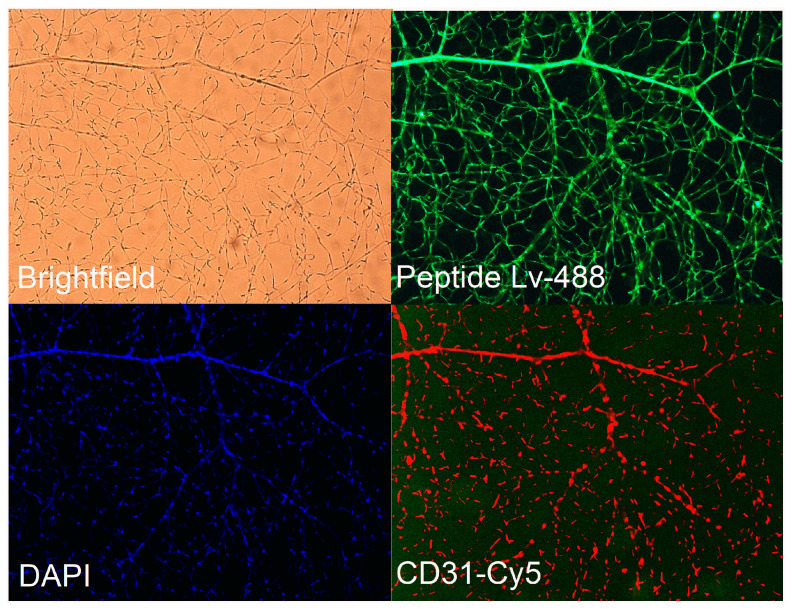
Peptide Lv is expressed in retinal vasculature. After trypsin digestion, the remaining retinal vasculature was stained with an antibody specific to peptide Lv (anti-Lv; green), endothelial marker CD31 (red), and DAPI (blue). Photos were taken under a 20× objective lens.

Previously, we showed that peptide Lv is expressed in the neural retina [3]. To confirm its expression in retinal vasculature, mice retinas were digested with trypsin to isolate retinal vasculature from the neural tissue followed by co-immunostaining with an antibody against peptide Lv (anti-Lv) and CD31, an endothelial marker. Retinal vascular ECs expressed peptide Lv, as peptide Lv was localized in retinal microvasculature that was labelled with CD31 (Figure 1).

**Figure 2:**
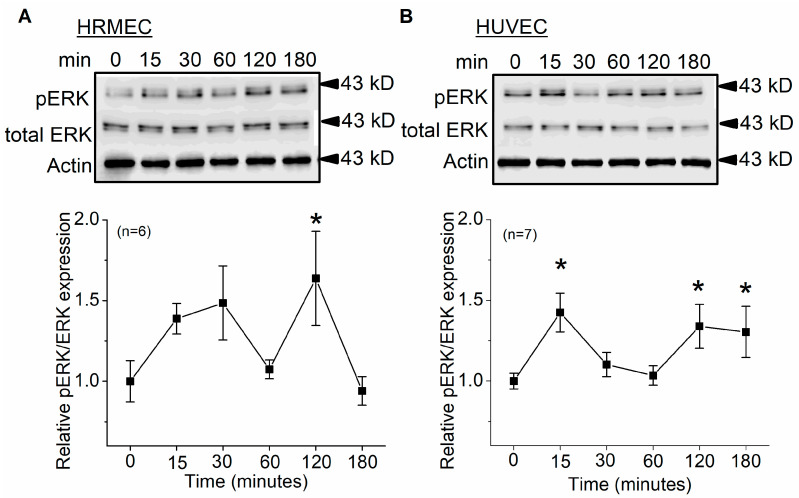
Peptide Lv elicits ERK phosphorylation in ECs. Cultured HRMECs and HUVECs were treated with peptide Lv for various durations (0, 15, 30, 60, 120, and 180 min). The Western blots show phosphorylated ERK (pERK) and total ERK in (**A**) HRMECs and (**B**) HUVECs. One-way ANOVA followed with Tukey *post hoc* tests were used for statistical analyses; * *p* < 0.05.

Since peptide Lv elicits ERK activation/phosphorylation in photoreceptors [1], we next determined whether exogenous peptide Lv also activated ERK in vascular ECs. Cultured HRMECs and HUVECs were treated with peptide Lv (500 ng/mL) for various periods of time and then harvested for Western blots. Treatment with peptide Lv for 120 min significantly elicited ERK phosphorylation in both HRMECs and HUVECs, but peptide Lv did not affect the total level of ERK in ECs (Figure 2). Activation of ERK signaling might be important in mediating the bioactivities of peptide Lv.

**Figure 3:**
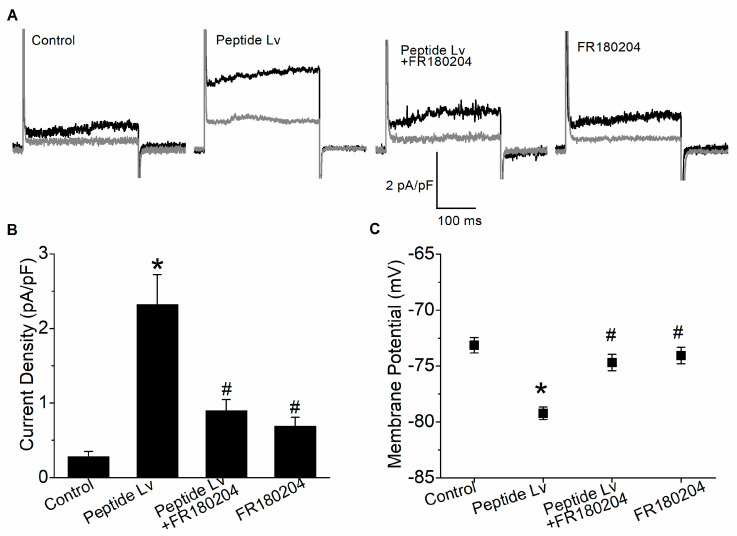
Blocking ERK activation attenuates peptide Lv-mediated increase in K_Ca_3.1 current densities and endothelial hyperpolarization. HUVECs were seeded onto glass coverslips and kept in an incubator for 48 h to allow the cells to adhere. The cultures were then treated with PBS (vehicle control), peptide Lv (500 ng/mL), FR180204 (ERK inhibitor; 10 μM), or peptide Lv and FR180204 for 3 h prior to whole-cell electrophysiological recordings. (**A**) Representative traces are displayed with the total outward current (black) and the current after perfusion with TRAM-34 to isolate K_Ca_3.1 currents (gray). (**B**) The current density (pA/pF) was obtained by dividing the K_Ca_3.1 current amplitude (measured at 200 ms; the tau point) by the whole-cell capacitance. Peptide Lv significantly increases the K_Ca_3.1 current densities, which was attenuated by FR180204. (**C**) Membrane potentials are significantly hyperpolarized in HUVECs treated with peptide Lv compared to the PBS-treated control, while treatment with FR180204 attenuates peptide Lv-elicited EC hyperpolarization. One-way ANOVA followed with Tukey *post hoc* tests were used for statistical analyses; *n* = 12–16 for each group; * *p* < 0.05. *: significantly different compared to the control; #: significantly different from the peptide Lv group.

Previously, we showed that peptide Lv hyperpolarizes cultured ECs by increasing K_Ca_3.1 current densities [4]. To determine whether the activation of ERK was needed for augmentation of K_Ca_3.1 and endothelial hyperpolarization by peptide Lv, we performed patch-clamp electrophysiological recordings on cultured HUVECs after 3 h of treatment of PBS (vehicle control) or peptide Lv (500 ng/mL) in the presence or absence of FR180204 (10 μM; an ERK inhibitor; Figure 3). Representative traces (Figure 3A) showed that, after an outward current (black) was recorded, TRAM-34 (K_Ca_3.1 inhibitor) was perfused followed by a second current recording (gray). The subtraction between these two currents indicates the isolated K_Ca_3.1 current. Peptide Lv significantly increased the K_Ca_3.1 current densities (2.32 ± 0.40 pA/pF) compared to the control (0.28 ± 0.07 pA/pF) or FR180204 treatment alone (0.69 ± 0.12 pA/pF; * *p* < 0.05; Figure 3A,B). Peptide Lv-elicited increases in K_Ca_3.1 were attenuated by ERK inhibition (peptide Lv + FR180204: 0.90 ± 0.15 pA/pF, # *p* < 0.05 compared to the peptide Lv group; Figure 3A,B). In addition, peptide Lv-induced hyperpolarization of HUVECs (−79.23 ± 0.56 mV compared to the control (−73.14 ± 0.69 mV) or HUVECs treated with FR180204 alone (−74.07 ± 0.74 mV); * *p* < 0.05; Figure 3C) was attenuated when ERK activation was blocked with FR180204 (−74.68 ± 0.72 mV; # *p* < 0.05 compared to the peptide Lv group; Figure 3C). Thus, peptide Lv-elicited augmentation of K_Ca_3.1 current densities and EC hyperpolarization are in part through ERK activation.

### 3.2. Peptide Lv Promotes K_Ca_3.1 Channel Trafficking and Membrane Insertion through the MEK1–ERK Signaling Pathway

**Figure 4:**
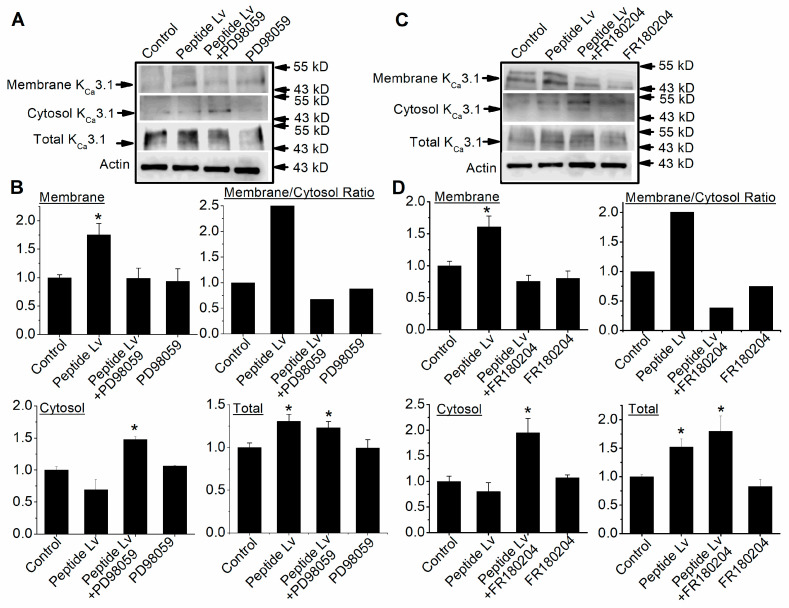
Blocking ERK activation decreases peptide Lv-elicited increase in membrane-bound K_Ca_3.1 without affecting the total protein expression of K_Ca_3.1. HUVEC cultures were treated with PBS (vehicle control) or peptide Lv (500 ng/mL) in the presence/absence of PD98059 (MEK1 inhibitor; 10 μM; (**A**,**B**)) or FR180204 (ERK inhibitor; 10 μM; (**C**,**D**)) for 3 h. Cell-surface biotinylation assays were performed on the cultures to separate the membrane-bound proteins from the cytoplasmic proteins. An aliquot of each sample was used for total protein analysis, and the remainder was used to separate the membrane-bound from cytosolic proteins. Actin was used as the loading control. The Y-axes of the “Membrane” (membrane-bound), “Cytosol” (cytosolic), and “Total” (total proteins) are presented as “Relative K_Ca_3.1/Actin”. (**A**,**B**) Peptide Lv significantly increases the total expression of K_Ca_3.1 as well as the membrane-bound K_Ca_3.1. PD98059 does not affect the total expression of K_Ca_3.1 but significantly decreases peptide Lv-elicited increase in membrane-bound K_Ca_3.1, which is also reflected as a significantly higher amount of cytosolic K_Ca_3.1. (**C**,**D**) Similarly, FR180204 does not affect the total expression of K_Ca_3.1 but significantly decreases peptide Lv-elicited increase in membrane-bound K_Ca_3.1. One-way ANOVA followed with Tukey *post hoc* tests were used for statistical analyses; *n* = 5 for each group; * *p* < 0.05.

After ion channel proteins are expressed, they need to be transported into the plasma membrane to be functional. The ERK signaling is known to promote ion channel trafficking and plasma membrane insertion in neurons [20]. As peptide Lv can elicit ERK activation/phosphorylation and increase K_Ca_3.1 current densities, we postulated that activated ERK mediates peptide Lv-elicited increases in K_Ca_3.1 trafficking in ECs. We performed biotinylation assays after HUVECs were treated for 3 h with peptide Lv in the absence or presence of PD98059 (50 μM; a MEK1 inhibitor) or FR180204 (10 μM; an ERK inhibitor) to determine if blocking MEK1–ERK signaling would prevent peptide Lv-stimulated K_Ca_3.1 trafficking and plasma membrane insertion. We previously showed that treatment with peptide Lv for 3 h increases both mRNA and protein expression in cultured RMECs and HUVECs [4]. We found that peptide Lv not only increased the total protein expression of KC_a_3.1 (* *p* < 0.05), but also indeed promoted K_Ca_3.1 insertion into the plasma membrane, as the level of membrane-bound K_Ca_3.1 was significantly higher in peptide Lv-treated ECs compared to the control (* *p* < 0.05; Figure 4). Blocking the activation of MEK1 (Figure 4A,B) or ERK (Figure 4C,D) significantly attenuated peptide Lv-elicited increases in membrane-bound K_Ca_3.1 (* *p* < 0.05; Figure 4). Interestingly, inhibition of MEK1–ERK signaling did not affect peptide Lv-elicited increases in the total protein level of K_Ca_3.1 (* *p* < 0.05). These data indicate that peptide Lv-stimulated increases in K_Ca_3.1 current densities (Figure 3) are in part through MEK1–ERK signaling-mediated protein trafficking and plasma membrane insertion of K_Ca_3.1 in ECs.

### 3.3. Peptide Lv Augments K_Ca_3.1 Current Density and Endothelial Hyperpolarization through Akt Activation

**Figure 5:**
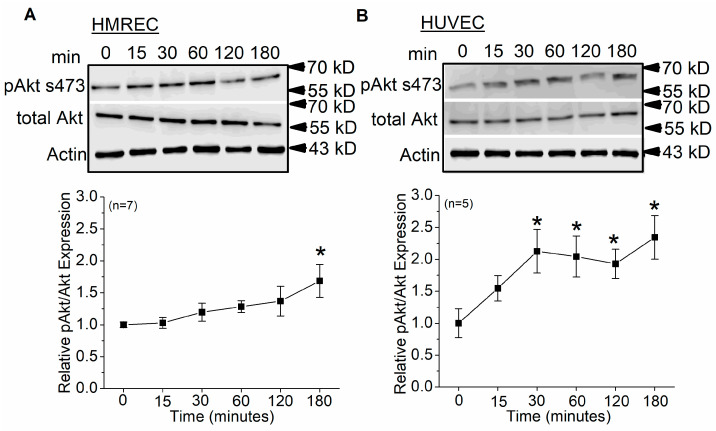
Peptide Lv elicits Akt phosphorylation/activation in ECs. Cultured HRMECs and HUVECs were treated with peptide Lv for various durations (0, 15, 30, 60, 120, and 180 min). The Western blots show phosphorylated Akt at ser473 and total Akt in (**A**) HMRECs and (**B**) HUVECs. One-way ANOVA followed with Tukey *post hoc* tests were used for statistical analyses; * *p* < 0.05.

The PI3K–Akt signaling pathway is another pathway known to promote ion channel trafficking and membrane insertion [18]. We found that HRMECs (Figure 5A) or HUVECs (Figure 5B) treated with peptide Lv (500 ng/mL) for 3 h had a significant increase in Akt phosphorylation at serine 473 (ser473), without affecting the total level of Akt.

**Figure 6:**
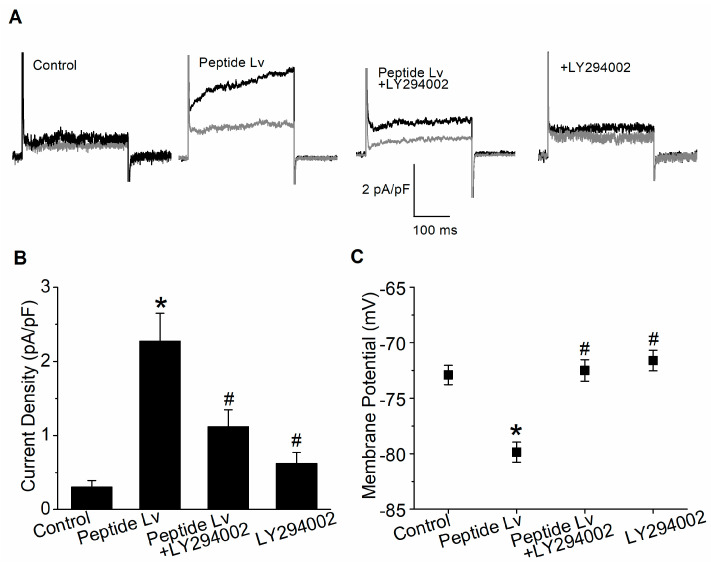
Blocking Akt activation attenuates peptide Lv-mediated increase in K_Ca_3.1 current densities and endothelial hyperpolarization. HUVECs were seeded onto glass coverslips and kept in an incubator for 48 h to allow the cells to adhere. The cultures were then treated with PBS (vehicle control), peptide Lv (500 ng/mL), LY294002 (PI3K inhibitor, 10 μM), or peptide Lv and LY294002 for 3 h prior to whole-cell electrophysiological recordings. (**A**) Representative traces are displayed with the total outward current (black) and the current after perfusion with TRAM-34 to isolate K_Ca_3.1 currents (grey). (**B**) The current density (pA/pF) was obtained by dividing the K_Ca_3.1 current amplitude (measured at 200 ms; the tau point) by the whole-cell capacitance. Peptide Lv significantly increases the K_Ca_3.1 current densities, which were attenuated by LY294002. (**C**) Membrane potentials are significantly hyperpolarized in HUVECs treated with peptide Lv compared to the PBS-treated control, while treatment with LY294002 significantly dampens peptide Lv-elicited EC hyperpolarization. One-way ANOVA followed with Tukey *post hoc* tests were used for statistical analyses; *n* = 12–16 for each group; * *p* < 0.05. *: significantly different compared to the control; #: significantly different from the peptide Lv group.

To determine if the activation of PI3K–Akt signaling was needed for augmentation of K_Ca_3.1 and endothelial hyperpolarization by peptide Lv, we performed patch-clamp electrophysiological recordings on cultured HUVECs after 3 h of treatment of PBS (vehicle control) or peptide Lv (500 ng/mL) in the presence or absence of LY294002 (50 μM; a PI3K inhibitor; Figure 6). Peptide Lv significantly increased the K_Ca_3.1 current densities (2.28 ± 0.37 pA/pF) compared to the control (0.31 ± 0.08 pA/pF) or LY294002 treatment alone (0.63 ± 0.14 pA/pF; * *p* < 0.05; Figure 6A,B). Peptide Lv-elicited increases in K_Ca_3.1 were attenuated by PI3K inhibition (peptide Lv + LY294002: 1.12 ± 0.22 pA/pF, # *p* < 0.05 compared to the peptide Lv group; Figure 6A,B). Peptide Lv-induced hyperpolarization of HUVECs (−79.86 ± 0.90 mV compared to the control (−72.90 ± 0.87 mV) or HUVECs treated with LY204992 alone (−71.61 ± 0.93 mV); * *p* < 0.05; Figure 6C), was attenuated when PI3K–Akt activation was blocked by LY294002 (−72.49 ± 0.96 mV; # *p* < 0.05 compared to the peptide Lv group; Figure 6C). Thus, peptide Lv-elicited augmentation of K_Ca_3.1 current densities and EC hyperpolarization are also mediated by PI3K–Akt signaling.

### 3.4. Peptide Lv Promotes K_Ca_3.1 Channel Trafficking and Membrane Insertion through the PI3K–Akt Signaling Pathway

**Figure 7:**
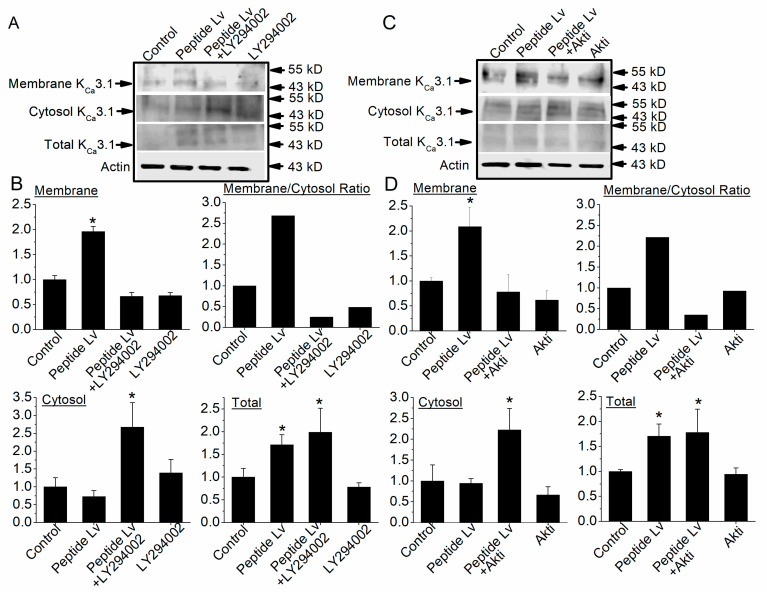
Blocking Akt activation decreases peptide Lv-elicited increase in membrane-bound K_Ca_3.1 without affecting the total protein expression of K_Ca_3.1. HUVEC cultures were treated with PBS (vehicle control) or peptide Lv (500 ng/mL) in the presence/absence of LY294002 (PI3K inhibitor; 10 μM; (**A**,**B**)) or Akti (Akt inhibitor; 10 μM (**C**,**D**)) for 3 h. Cell-surface biotinylation assays were performed on the cultures to separate the membrane-bound proteins from the cytoplasmic proteins. After sample collection, an aliquot of each was used for total protein analysis, and the remainder was used to separate the membrane-bound from cytosolic proteins. Actin was used as the loading control. The Y-axes of the “Membrane” (membrane-bound), “Cytosol” (cytosolic), and “Total” (total proteins) are presented as “Relative K_Ca_3.1/Actin”. (**A**,**B**) Peptide Lv significantly increases the total expression of K_Ca_3.1 as well as the membrane-bound K_Ca_3.1. LY294002 does not affect the total expression of K_Ca_3.1 but significantly decreases peptide Lv-elicited increase in membrane-bound K_Ca_3.1, which is also reflected as a significantly higher amount of cytosolic K_Ca_3.1. (**C**,**D**) Similarly, Akti does not affect the total expression of K_Ca_3.1 but significantly decreases peptide Lv-elicited increase in membrane-bound K_Ca_3.1. One-way ANOVA followed with Tukey *post hoc* tests were used for statistical analyses; *n* = 5 for each group; * *p* < 0.05.

We next employed biotinylation assays to determine whether PI3K–Akt signaling also mediates peptide Lv-elicited channel trafficking and plasma membrane insertion of K_Ca_3.1. Cultured HUVECs were treated for 3 h with PBS (vehicle control) or peptide Lv (500 ng/mL) with or without the presence of LY294002 (50 μM) or Akti (10 μM; an Akt inhibitor). We found that blocking the activation of PI3K (Figure 7A,B) or Akt (Figure 7C,D) significantly attenuated peptide Lv-elicited increases in membrane-bound K_Ca_3.1 (* *p* < 0.05; Figure 7). In addition, inhibition of PI3K–Akt signaling did not affect peptide Lv-elicited increases in K_Ca_3.1 total protein (* *p* < 0.05). These data indicate that peptide Lv-stimulated increases in K_Ca_3.1 current densities (Figure 6) are in part through PI3K–Akt signaling-mediated protein trafficking and plasma membrane insertion of K_Ca_3.1 in ECs.

### 3.5. Peptide Lv Concurrently Activates the MEK1–ERK and PI3K–Akt Signaling Pathways

**Figure 8:**
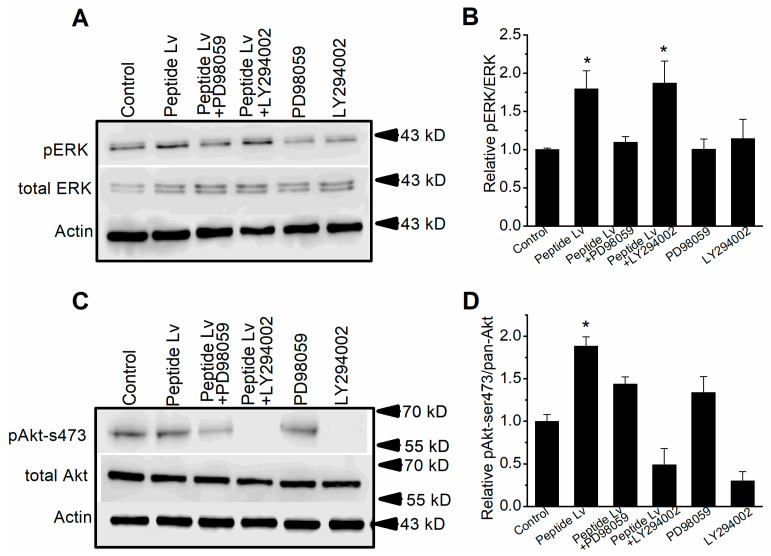
Peptide Lv concurrently activates the MEK1–ERK and PI3K–Akt signaling pathways. Immunoblot assays were performed on HUVEC cultures treated with PBS (vehicle control) or peptide Lv (500 ng/mL) in the presence/absence of FR180204 (ERK inhibitor) or LY294002 (PI3K inhibitor). (**A**,**B**) Peptide Lv increases phosphorylated ERK (pERK), which is attenuated by FR180204 but not LY294002. (**C**,**D**) Peptide Lv increases phosphorylated Akt at ser473, which is attenuated by LY294002 but not FR18204. One-way ANOVA followed with Tukey *post hoc* tests were used for statistical analyses; *n* = 7 for each group; * *p* < 0.05.

Since both MEK1–ERK and PI3K–Akt signaling pathways mediated peptide Lv-elicited increases in K_Ca_3.1 current densities in ECs, we next determined the possible interactions between these two signaling pathways and whether one was upstream/downstream to the other. We treated HUVECs for 3 h with PBS (vehicle control) or peptide Lv (500 ng/mL) in the presence or absence of an inhibitor of MEK1 (PD98059; 50 μM), ERK (FR180204; 10 μM), PI3K (LY294002; 50 μM), or Akt (Akti; 10 μM) to determine the levels of phosphorylated ERK and Akt. As peptide Lv activated/phosphorylated ERK (Figure 8A,B) and Akt (Figure 8C,D), inhibition of MEK1 prevented peptide Lv-stimulated activation of ERK without affecting Akt phosphorylation at Ser473. Similarly, inhibition of PI3K prevented peptide Lv-stimulated phosphorylation of Akt without affecting ERK phosphorylation. These findings suggest that peptide Lv can independently and concurrently activate MEK1–ERK and PI3K–Akt to mediate peptide Lv-elicited increases in K_Ca_3.1 and its trafficking. 

## 4. Discussion

Previously, we showed that peptide Lv enhances K_Ca_3.1 current densities in vascular ECs to promote angiogenesis [4], but the mechanism by which these currents are enhanced was unclear. The current densities of ion channels can be augmented through various mechanisms, including posttranslational modifications [11,27,28], stimulating trafficking and insertion into the plasma membrane [18,20], or preventing internalization and degradation of the channel proteins [19,29,30].

Peptide Lv increases the protein expression and the current densities of endothelial K_Ca_3.1 channels after ECs were treated with peptide Lv for 3 h [4], so it is likely that peptide Lv promotes the trafficking and insertion of K_Ca_3.1 into the plasma membrane. The MEK1–ERK and PI3K–Akt signaling pathways are known to promote the trafficking and insertion of ion channels into the plasma membrane in neurons [18,20]. We found that peptide Lv stimulated both pathways in two different types of ECs, HRMECs and HUVECs (Figure 2 and Figure 5), but the timing of peptide Lv-elicited activation of ERK and Akt were different in these two cell types: HUVECs appeared to have earlier activations of peptide Lv-elicited phosphorylation of ERK and Akt compared to that of HRMECs. This could be due to the different properties of ECs in large vessels (HUVECs) versus in microvasculature (HRMECs), and ERK and Akt were activated after these ECs treated with peptide Lv for less than 3 h. This indicates that peptide Lv elicited ERK and Akt activations first, and these two pathways further stimulated K_Ca_3.1 trafficking onto the plasma membrane, so the timing of ERK and Akt activation was associated with the timing of peptide Lv-elicited increase in K_Ca_3.1 current densities. Blocking these signaling pathways prevented peptide Lv-elicited increase in K_Ca_3.1 current densities (Figure 3 and Figure 6). While pharmacological inhibitors can have nonspecific effects, we employed a strategy of using two different inhibitors with different mechanisms of action in a single signaling pathway to confirm these results in our study. Interestingly, blocking these pathways only prevented peptide Lv-mediated increase in membrane-bound K_Ca_3.1 but not the overall increase in K_Ca_3.1 expression (Figure 4 and Figure 7). These findings suggest that the MEK1–ERK and PI3K–Akt signaling pathways are responsible for peptide Lv-elicited increases in K_Ca_3.1 current densities by promoting trafficking and membrane insertion of the channels, but they do not affect the overall protein expression. Since we performed these experiments in monolayer cultured ECs, these data might not be exactly the same as ECs in the three-dimensional environment in vivo, which is a limitation of this study. As we showed that peptide Lv elicits neovascularization in vivo [3], and endothelial K_Ca_3.1 is required for EC-dependent neovascularization [14], nonetheless, these data in part reflect the molecular mechanism as to how peptide Lv elicits EC-dependent neovascularization through promoting K_Ca_3.1 trafficking onto the plasma membrane. 

These findings do not eliminate the possibility that peptide Lv can enhance K_Ca_3.1 current densities through other mechanisms including posttranslational modulation. For example, phosphorylation of K_Ca_ channels can enhance its currents [20]. Previously, we showed that peptide Lv can elicit vasodilation in isolated arterioles after the vessels were bathed with a solution containing peptide Lv (1–10 μg/mL) for 2 or 3 min [3]. This finding suggests that peptide Lv may acutely elicit posttranslational modifications through activation of kinases, which then phosphorylates and opens K_Ca_3.1 channels and leads to hyperpolarization of vascular ECs and vasodilation. As we stated earlier that peptide Lv-elicited vasodilation is partially NO-independent, further studies are needed to determine how peptide Lv might elicit posttranslational modulation of K_Ca_3.1. 

Previously, we found that peptide Lv increases cyclic adenosine monophosphate (cAMP) levels in cultured photoreceptors [1]. Cyclic AMP signaling is known to activate protein kinase A (PKA) [31], and the c-terminal phosphorylation of K_Ca_3.1 by cAMP/PKA increases the channel activity [32,33], which could be a potential signaling pathway mediating the acute action of peptide Lv on vasodilation. Peptide Lv does not have any kinase activity, making it unlikely that peptide Lv directly activates the MEK1–ERK or PI3K–Akt signaling pathways. Further studies are needed to elucidate the upstream signaling of MEK1–ERK and PI3K–Akt activated by peptide Lv, whether acutely or chronically.

In addition to ion channel trafficking, the MEK1–ERK and PI3K–Akt signaling pathways are associated with angiogenesis through various processes [34,35,36,37]. Blocking ERK signaling impaired EC proliferation, migration, and sprouting [38,39]. EC migration and vessel tube formation is PI3K–Akt-dependent [40,41]. Knockout of Akt decreased EC proliferation, migration, and sprouting [42,43]. Both pathways are also downstream signaling of VEGF receptor 2 (VEGFR2) [44,45,46]. As we previously showed that peptide Lv is able to bind VEGFR2 [2], it is also possible that peptide Lv concurrently activates ERK and Akt signaling in part through binding VEGFR2, which can lead to EC proliferation, migration, and sprouting, and neovascularization [3].

## 5. Conclusions

This study provides further insights into the molecular mechanisms of peptide Lv in augmenting EC functions. Blocking peptide Lv may become a novel therapy to combat diseases associated with pathological angiogenesis, as we previously showed that using an antibody against peptide Lv blocked pathological neovascularization [3]. By contrast, perhaps peptide Lv may be used in situations that require angiogenesis, such as wound healing and recovery from ischemia. Previously, we showed that peptide Lv works synergistically with VEGF to promote EC proliferation [3], which may explain why a single dose of anti-VEGF therapy cannot block recurring neovascularization. Delineating the molecular mechanism of bioactivity of peptide Lv is important to combat diseases with pathological neovascularization.

In addition to promoting angiogenesis and vasodilation, peptide Lv may have other unknown functions. Peptide Lv is a widely expressed secretory peptide that has been shown to be important in early photoreceptor development and cardiomyocyte function [1,2]. Peptide Lv may also play a role in immune responses, as it dampens the inflammatory response in macrophages [47]. Much of the function and mechanism of peptide Lv remains unknown and should be investigated.

## Data Availability

All data related to this study are presented in this manuscript.
